# A Model for Developing Subspecialty Clinical Practice Guidelines: The Geriatric Emergency Department Guidelines 2.0

**DOI:** 10.1016/j.acepjo.2025.100247

**Published:** 2025-09-16

**Authors:** Satheesh Gunaga, Christopher R. Carpenter, Maura Kennedy, Lauren T. Southerland, Alexander X. Lo, Sangil Lee, Kirby Swan, Fabrice Mowbray, Rachel M. Skains, Teresita M. Hogan, Martin F. Casey, Kei Ouchi, Naomi R. George, Kerstin de Wit, Cameron J. Gettel, Katherine Selman, Luna C. Ragsdale, Anita N. Chary, James D. van Oppen, Glenn Arendts, Charles L. Maddow, Katherine M. Hunold, Katren R. Tyler, Danya Khoujah, Ula Hwang, Shan Liu

**Affiliations:** 1 Department of Emergency Medicine, Henry Ford Health, Wyandotte Hospital, Wyandotte, Michigan, USA; 2 Envision Healthcare, Ann Arbor, Michigan, USA; 3 Department of Osteopathic Medical Specialties, Michigan State University College of Osteopathic Medicine, East Lansing, Michigan, USA; 4 Department of Emergency Medicine, Mayo Clinic, Rochester, Minnesota, USA; 5 Department of Emergency Medicine, Massachusetts General Hospital, Boston, Massachusetts, USA; 6 Department of Emergency Medicine, Harvard Medical School, Boston, Massachusetts, USA; 7 Department of Emergency Medicine, The Ohio State University, Columbus, Ohio, USA; 8 Department of Emergency Medicine, Northwestern University Feinberg School of Medicine, Chicago, Illinois, USA; 9 Department of Emergency Medicine, University of Iowa Carver College of Medicine, Iowa City, Iowa, USA; 10 College of Nursing & College of Human Medicine, Michigan State University, East Lansing, Michigan, USA; 11 Department of Emergency Medicine, University of Alabama at Birmingham, Birmingham, Alabama, USA; Geriatric Research, Education and Clinical Center, Birmingham VAMC, Birmingham, Alabama, USA; 12 Section of Emergency Medicine, Department of Medicine, University of Chicago Pritzker School of Medicine, Chicago, Illinois, USA; 13 Department of Emergency Medicine, University of North Carolina School of Medicine, Chapel Hill, North Carolina, USA; 14 Department of Emergency Medicine, Brigham and Women's Hospital, Boston, Massachusetts, USA; 15 Department of Emergency Medicine, Division of Critical Care, University of New Mexico School of Medicine, Albuquerque, New Mexico, USA; 16 Department of Emergency Medicine, Queens University, Kingston, Ontario, Canada; 17 Department of Emergency Medicine, Yale University, New Haven, Connecticut, USA; 18 Center for Outcomes Research and Evaluation, Yale School of Medicine, New Haven, Connecticut, USA; 19 Department of Emergency Medicine, Cooper Medical School of Rowan University, Camden, New Jersey, USA; 20 Department of Emergency Medicine, Durham VA Health Care System, Durham, North Carolina, USA; 21 Department of Emergency Medicine, Duke University School of Medicine, Durham, North Carolina, USA; 22 Department of Emergency Medicine, Medicine-Section of Health Services Research, Baylor College of Medicine, Houston, Texas, USA; 23 Centre for Urgent and Emergency Care Research, University of Sheffield, Sheffield, United Kingdom; 24 University of Western Australia Medical School, Crawley, Western Australia, Australia; 25 Department of Emergency Medicine, University of Texas Health Science Center at Houston McGovern Medical School, Houston, Texas, USA; 26 Department of Emergency Medicine, University of California Davis School of Medicine, Sacramento, California, USA; 27 Department of Emergency Medicine, University of Maryland School of Medicine, Baltimore, Maryland, USA; 28 Geriatric Research, Education and Clinical Center, James J. Peters VAMC, Bronx, New York, USA; 29 Department of Emergency Medicine and Population Health, NYU Grossman School of Medicine, New York, USA

**Keywords:** emergency medicine, geriatrics, aged, evidence-based medicine, practice guidelines as topic, health services for the aged, program development

## Abstract

The original consensus–based Geriatric Emergency Department (GED) Guidelines, published in 2014, established a framework of core principles for delivering high-quality, age-appropriate emergency care for older adults. In response to significant advances in geriatric emergency medicine research and evolving clinical priorities, we developed the GED Guidelines 2.0 to ensure continued relevance, clinical utility, and evidence-based rigor. This concept paper describes the systematic and iterative process undertaken to update the guidelines, including the formation of multidisciplinary working groups and the application of the Grading of Recommendations Assessment, Development, and Evaluation (GRADE) methodology. Unlike the original GED Guidelines, our approach prioritized methodological transparency, formalized evidence grading, and consensus building grounded in systematic reviews and meta-analyses. We describe the identification, recruitment, and collaboration of multidisciplinary clinical and academic experts working together to improve the care of older adults in the emergency department. Through this multidisciplinary effort, key geriatric domains were selected, priority topics identified, and systematic reviews and meta-analyses conducted to generate a robust evidence base for future guideline and policy development. The GED Guidelines 2.0 represents the first emergency medicine (EM) subspecialty guideline effort to fully adopt the GRADE framework, offering a novel blueprint for future EM guideline development.

## 1 Background

### 1.1 A Brief History of Geriatric Emergency Medicine

**Figure 1:**
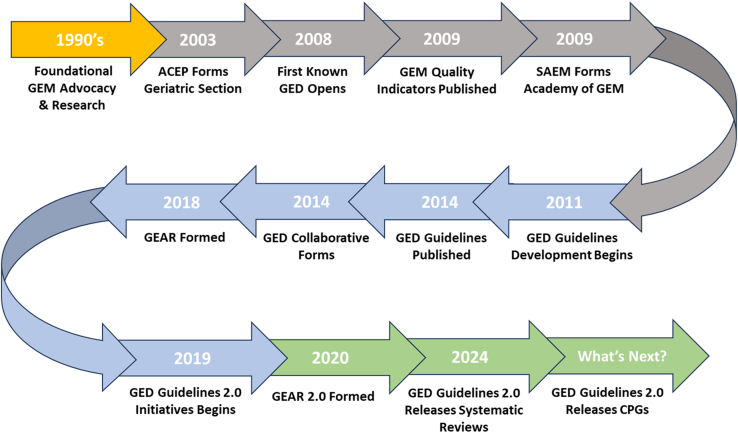
Timeline of key milestones in geriatric emergency medicine, highlighting significant advancements related to the Geriatric Emergency Department Guidelines. ACEP, American College of Emergency Physicians; CPG, clinical practice guideline; GEAR, Geriatric Emergency Care Applied Research; GED, geriatric emergency department; GEM, geriatric emergency medicine; SAEM, Society for Academic Emergency Medicine.

Adults aged ≥65 years have the highest rates of emergency department (ED) visits and noncritical hospitalizations among all age groups in the United States (US).^1^^,^^2^ The process, experience, and outcomes of emergency care for older adults are uniquely complex due to geriatric syndromes, multimorbidity, and polypharmacy, which increase the risk of hospitalization and complicate care transitions.^3^, ^4^, ^5^ Older adults often present with additional challenges such as functional and cognitive impairment, social isolation, and unclear goals of care, which traditional ED workflows are not designed to address.^6^^,^^7^ For example, standard ED processes prioritize medical acuity and quick diagnosis, often overlooking the need for detailed medication reconciliation or consideration of frailty in decision making.^6^^,^^8^ Older adults may also require tailored discharge planning accounting for mobility limitations or cognitive decline; however, this is often not integrated into typical ED protocols.^9^^,^^10^ These gaps in care are crucial because they can lead to poor patient-centered outcomes, such as unnecessary admissions or post-ED functional decline.^3^^,^^4^^,^^6^^,^^7^ Decades of research and advocacy in geriatric emergency medicine (GEM) have driven the development of specialized care models to improve both patient and system outcomes (Fig 1).^11^ This momentum led to the establishment of the first self-described geriatric EDs (GEDs) in the US in 2008, and by 2013, 30 EDs had self-identified as GEDs, although care models varied significantly across institutions.^12^^,^^13^

### 1.2 Establishing the GED Guidelines: Origins, Scope, and Influence

Recognizing the need for standardized guidance to address the unique challenges of older adults in the ED, work on the original GED Guidelines began in 2011 and culminated in their publication in 2014.^14^, ^15^, ^16^ Developed through a consensus building effort with multidisciplinary stakeholders, including the American College of Emergency Physicians (ACEP), American Geriatrics Society, Emergency Nurses Association, and the Society for Academic Emergency Medicine (SAEM), the guidelines were designed to improve care and outcomes for older adults in emergency settings.^14^, ^15^, ^16^, ^17^ This effort marked a significant step forward in GEM, providing clear, evidence-based recommendations to optimize ED care delivery for older adults.^12^^,^^17^^,^^18^

The original GED Guidelines comprise 42 recommendations organized into 6 key categories: staffing, transitions of care, education, quality improvement, equipment and supplies, and policies, procedures, and protocols.^14^, ^15^, ^16^ Core recommendations emphasize geriatric-specific leadership roles, structured-discharge planning, integration with community resources, age-friendly ED design, and the use of evidence-based protocols for common geriatric presentations (Table 1).^14^^,^^19^ ACEP accredits GEDs based on adherence to best practices outlined in the GED Guidelines, with 541 EDs now accredited.^18^^,^^20^ Beyond accreditation, the guidelines have stimulated broader advancements in research, education, and care models within GEM.^21^, ^22^, ^23^, ^24^ For example, the guidelines have led to the development of targeted–geriatric training programs for ED staff, the implementation of protocols for delirium screening, and the creation of performance metrics to monitor outcomes like ED length of stay and 30-day readmission rates.^23^, ^24^, ^25^, ^26^ These innovations have fostered continuous improvement and laid the foundation for future guideline updates.

**Table 1:** Overview of the original geriatric emergency department Guidelines and the anticipated impact of the geriatric emergency department Guidelines 2.0.

6 general categories of recommendations	Original geriatric ED guidelines 42 specific recommendations	Geriatric ED Guidelines 2.0 7 GEM clinical practice guidelines
Staffing	Three recommendations ensure geriatric-trained physician and nursing leadership, including a GED medical director completing >8 h of geriatric CME every 2 y.	No anticipated new guidance.
Transitions of care	Four recommendations establish transition-of-care protocols for timely communication of geriatric-specific clinical information and maintaining community resource connections for seamless ED-to-outpatient transitions.	The GED Guidelines 2.0 clinical practice guidelines anticipate providing guidance on best practices around ED-to-outpatient transitions of care in older adults individualized for specific recommendations for delirium, dementia, falls, frailty, medication management, palliative care, and elder abuse which incorporate patient values and shared decision making.
Education	Three recommendations enhance continuing medical education programs to improve physician and nursing staff awareness of geriatric emergency care needs, policies, and procedures.	The GED Guidelines 2.0 anticipate supporting and building on current recommendations through a dissemination and implementation strategies that are individualized for each recommendation, cognizant of resource requirements and potential health inequities, and targeted to overcome anticipated obstacles to scaling up the intervention.
Quality improvement	Three recommendations require a geriatric quality-improvement program, overseen by the GED medical director and nurse manager.	The GED Guidelines 2.0 anticipate guiding future GED quality improvement programs by specifying numerator and denominators of target patient populations for guideline recommendations based on high certainty evidence.
Equipment and supplies	Four recommendations ensure ED physical infrastructure accommodates patients with mobility, continence, sensory, or cognitive impairments.	No anticipated new guidance.
Policies, procedures, and protocols	Geriatric ED screening (5 recommendations) Indwelling catheter (3 recommendations) Medication management (5 recommendations) Fall assessment (5 recommendations) Delirium and dementia (5 recommendations) Palliative care (2 recommendations)	7 New GRADE Level CPGs Delirium Dementia Falls Frailty Medication management Palliative care Elder abuse

CPG, clinical practice guidline; ED, emergency department; GED, geriatric emergency department; GEM, geriatric emergency medicine; GRADE, Grading of Recommendations Assessment, Development, and Evaluation.

### 1.3 Evolving Evidence and Expectations: The Justification for the GED Guidelines 2.0

Alongside the GED Guidelines, significant GEM progress has been driven by the Geriatric Emergency care Applied Research (GEAR) Network and its successor, GEAR 2.0.^9^^,^^27^, ^28^, ^29^, ^30^, ^31^ Like its pediatric counterpart, the Pediatric Emergency Care Applied Research Network, the GEAR initiatives have identified and funded critical research addressing key gaps in geriatric emergency care.^9^^,^^10^^,^^27^, ^28^, ^29^, ^30^, ^31^, ^32^, ^33^ These efforts targeted areas such as transitions of care, cognitive impairment, elder abuse, falls, medication safety, and dementia. The resulting growth in evidence motivated the update to the GED Guidelines to ensure they reflect current evidence-based recommendations.

Another key reason for the update is that the original guidelines were developed without a formal assessment of the quality, quantity, reproducibility, or applicability of the supporting evidence.^34^^,^^35^ Since that time, the expectations for clinical practice guidelines have evolved. Current guideline standards now require rigorous assessment of direct and indirect evidence, as well as transparent consideration of research bias, stakeholder values, health equity, anticipated costs, and clearly defined patient-centered outcomes, including the balance of anticipated benefits and potential harms.^35^, ^36^, ^37^, ^38^

Although the original GED Guidelines have had a significant impact on accredited GEDs (which represent <10% of US EDs), many EDs still face substantial implementation barriers due to the absence of local champions, competing clinical priorities, limited resources, and growing financial constraints.^39^, ^40^, ^41^ For many ED teams, the guidelines represent an ideal vision of geriatric emergency care that remains difficult to achieve without institutional and interdisciplinary support.^39^^,^^42^^,^^43^ Furthermore, many emergency medicine (EM) clinicians outside the GED-accredited EDs may have minimal awareness of the guidelines or their relevance to daily GEM practice, which has hindered broader dissemination and adoption.^23^^,^^44^

To address these challenges and evolving standards, the GED Guidelines 2.0 initiative launched in 2019 with 3 key objectives: (1) to update the evidence base supporting recommendations, (2) to enhance transparency and usability, and (3) to develop improved dissemination and implementation strategies. A major emphasis of the GED Guidelines 2.0 was to ensure practical usability, empowering EM clinicians to integrate evidence-based care for older adults in both accredited and non–accredited GED settings, with recommendations tailored to diverse resource levels and care environments.

**Figure 2:**
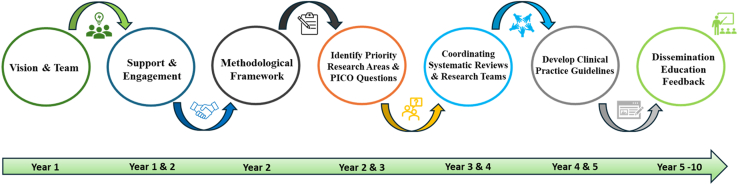
A roadmap for developing the Geriatric Emergency Department Guidelines 2.0.

**Figure 3:**
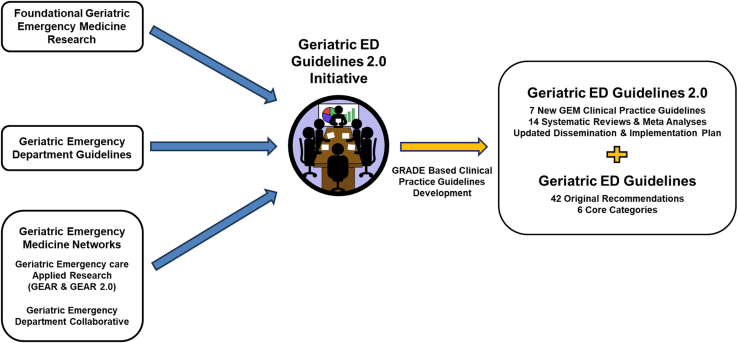
Geriatric Emergency Department Guidelines 2.0: Visual summary of input & impact. ED, emergency department; GRADE, Grading of Recommendations Assessment, Development, and Evaluation; GEM, geriatric emergency medicine.

As the first EM subspecialty group to adopt the Grading of Recommendations Assessment, Development, and Evaluation (GRADE) methodology for guideline development, the GED Guidelines 2.0 initiative aims to provide a transparent and replicable framework that can serve as a model for other EM subspecialty groups (Fig 2). Still in progress, the initiative will ultimately deliver 14 systematic reviews and meta-analyses (SRMAs) and 7 new GRADE–based clinical practice guidelines. These forthcoming guidelines will build on the original GED recommendations, offering structured, evidence-based guidance for high-priority geriatric conditions that are applicable across a wide spectrum of EDs (Fig 3, Table 1).

### 1.4 Launching the GED Guidelines 2.0 Initiative: Integrating Multidisciplinary Collaboration and GRADE Methodology

In 2019, the GED Guidelines 2.0 initiative began with a small planning group and quickly expanded into a diverse, interdisciplinary collaboration of >60 members. Participants included EM physicians, geriatricians, nurses, and allied health professionals from 23 US states and 7 countries, each contributing valuable expertise on clinical care delivery. To ensure broad applicability, the working group actively engaged national and international EM and geriatric organizations. In addition, several patient caregivers participated in the initiative, offering essential insights into patient needs, values, and care preferences.^45^

**Figure 4:**
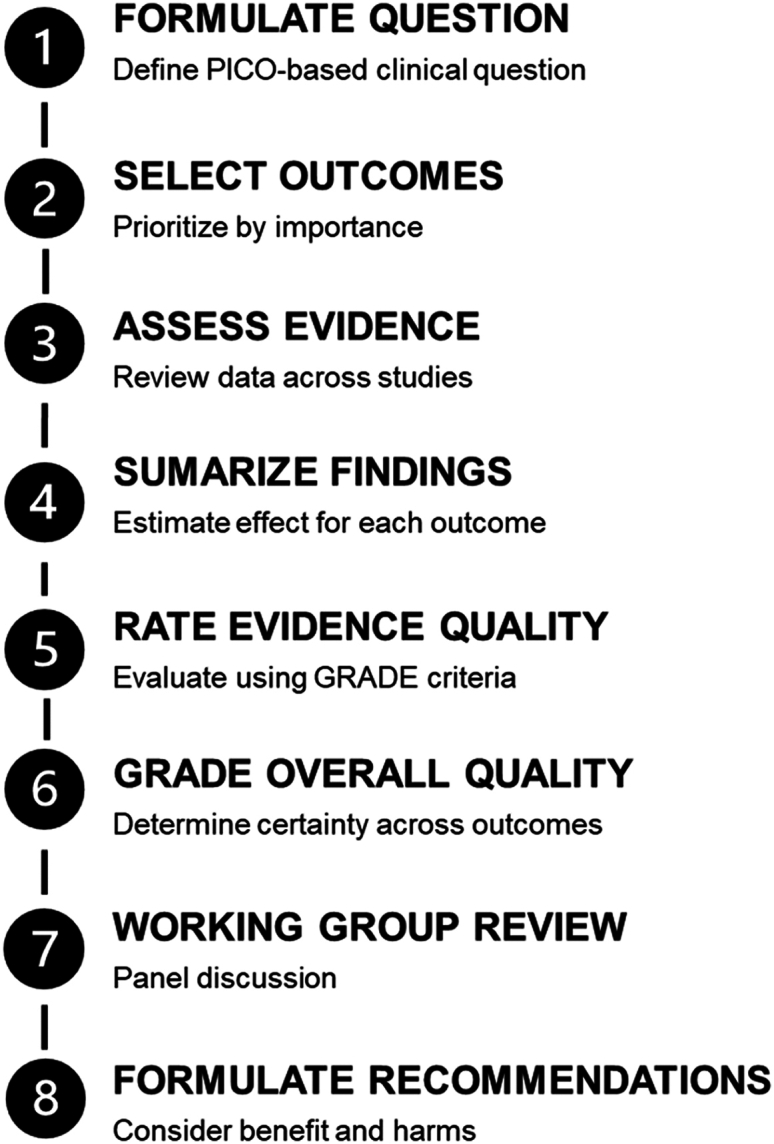
Overview of the Grading of Recommendations Assessment, Development, and Evaluation (GRADE) methodology. PICO, Population, Intervention, Comparator, and Outcomes.^46^

As the initiative progressed, the group undertook a careful evaluation of guideline development methodologies. By January 2021, following extensive discussion and GRADE training sessions, the working group formally voted to follow the GRADE framework.^36^^,^^46^ Recommended by methodologic experts for its rigor, transparency, and international alignment, GRADE has been widely used by organizations such as the World Health Organization and the Agency for Healthcare Research and Quality.^35^, ^36^, ^37^, ^38^^,^^46^, ^47^, ^48^, ^49^, ^50^, ^51^, ^52^, ^53^, ^54^, ^55^, ^56^, ^57^ GRADE offers a structured process for synthesizing evidence, framing research questions, and assessing bias, helping ensure that recommendations are both scientifically sound and clearly communicated (Fig 4).^46^

Although the group valued GRADE’s strengths, its resource-intensive nature was also recognized. Implementing GRADE requires trained methodologists, support from research librarians, and completion of systematic reviews and meta-analyses (SRMAs)—a considerable undertaking for a largely volunteer-driven initiative. Nevertheless, the group recognized that GRADE’s rigor would support the long-term usability of the GED Guidelines 2.0.

### 1.5 Identifying Priority Topics and Establishing GED Guidelines 2.0 Subcommittees

To begin the update process, the GED Guidelines 2.0 group first identified which original recommendations contained critical gaps in need of revision. Foundational elements such as interdisciplinary staffing, GED equipment and supplies, and educational requirements remained current and did not require formal revision. Building on this foundation, the group identified 7 high-priority clinical domains: frailty, medication safety, dementia, fall assessment and management, delirium, palliative care, and elder abuse.^9^^,^^14^, ^15^, ^16^^,^^28^, ^29^, ^30^^,^^32^^,^^58^ These areas were selected for their clinical significance, relevance to geriatric emergency care, and growing evidence base that could support the development of actionable recommendations.

Subcommittees were established for each priority topic, and members used the Population, Intervention, Comparator, Outcomes (PICO) framework to formulate structured clinical questions, guide literature searches, and inform evidence synthesis plans.^59^^,^^60^ The resulting PICO questions addressed a broad array of clinical issues, including screening protocols, diagnostic accuracy of assessment tools, imaging and laboratory evaluations, management strategies, and transitions of care. Critically, they also included outcomes aligned with current standards in clinical practice guideline development, such as functional status, quality of life, healthcare utilization, safety, and patient-centered outcomes. Table 2 summarizes the 14 finalized GED Guidelines 2.0 PICO questions, each designed to inform evidence-based recommendations that reflect the needs and priorities of older adults in emergency care settings.^61^, ^62^, ^63^, ^64^, ^65^, ^66^, ^67^, ^68^

**Table 2:** Description of the Geriatric Emergency Department Guidelines 2.0 priority topics and PICO questions.

Priority topics	PICO questions
Delirium	1a. In the ED, for all older adults aged ≥65 y, does using risk stratification to determine who should be screened for delirium improve prognostic accuracy, screening efficiency, and patient-oriented outcomes compared to not using a risk stratification method?^61^
	1b. In the ED, for all older adults aged ≥65 y, does the use of a risk stratification method to identify a subset of patients at higher risk for delirium improve the understanding of delirium prevalence and the proportion of older adults needing screening compared to not applying such a method?^61^
	2. In adults aged ≥60 y evaluated in the ED, how effective are various diagnostic approaches—including history, physical examination, laboratory testing, and screening instruments—in identifying delirium compared to an acceptable criterion standard for delirium in terms of diagnostic accuracy?^62^
	3. In patients aged ≥65 y with delirium who underwent head imaging in the ED, do abnormal neurologic examinations, headache, trauma, and anticoagulation identify acute abnormalities on head imaging (MRI/CT) as a possible or likely etiology of delirium?^63^^,^^64^
Falls	1. In adults aged ≥60 y presenting to the ED for a fall, how effective is a multifactorial fall prevention intervention compared to usual care in improving functional status, enhancing outpatient fall prevention, quality of life, reducing unscheduled healthcare use, and determining the new need for skilled care at discharge?
	2. In adults aged ≥60 y evaluated in the ED, how effective is evaluation by a therapist (physical, occupational, or both) for fall prevention, fall assessment, or mitigation of fall risk factors compared to no therapist evaluation in terms of the incidence of falls and other secondary patient-centered outcomes.
Medication safety	1. In older adults aged ≥65 y presenting with acute, undifferentiated agitation in the ED or out-of-hospital settings, which sedating medications are associated with the least adverse events?^65^
	2. Among older adults aged ≥65 y in the ED, how do various ED-based geriatric medication programs compare to traditional prescribing methods in their effectiveness at decreasing the rates of potentially innapropriate medications and adverse drug events?^66^
Frailty	1. In patients aged ≥65 y presenting to the ED, how does the use of a frailty assessment tool inform clinical decision making at triage or during the ED visit to improve patient outcomes compared to standard care?^67^
Dementia	1. In older persons living with dementia who have impaired cognition, what ED interventions improve patient-centered outcomes compared to usual care?^68^
	2. In older persons living with dementia who have impaired cognition, does the use of innovative pain assessment tools, compared to usual care, lead to more accurate or improved pain assessment in the ED?^68^
Palliative care	1. In older adults (≥60 y) with serious illness who present to the ED, is ED-based, palliative care screening associated with improved patient and health system outcomes when compared to those who do not receive screening in the ED?
	2. In older adults (≥60 y) with serious illness who present to the ED, is hospice and/or palliative care consultation initiated in the ED associated with improved patient and health system outcomes when compared to those who do not have consultation initiated in the ED?
Elder abuse	1. In ED patients aged ≥60 y, does universal or targeted screening for elder abuse, compared to usual care (clinical identification based on EMS, nurse, and physician gestalt and standard practice), improve the total number of cases identified, diagnostic accuracy, and long-term safety outcomes, including potential harms, legal outcomes, functional outcomes, psychosocial outcomes, and healthcare utilization?
	2. In ED patients aged ≥60 y who are previously known, newly found, or suspected victims of elder abuse, how do ED-based or ED-initiated interventions—including adult protective services reporting—compared with usual care in improving short-and long-term safety, health, legal, functional, and psychosocial outcomes?

CT, computed tomography; ED, emergency department; EMS, emergency medical services; MRI, magnetic resonance imaging; PICO, Population, Intervention, Comparator, Outcomes.

### 1.6 Creating Systematic Reviews, Meta-Analyses, and GRADE–Based Clinical Practice Guidelines

To support the GED Guidelines 2.0, PICO-specific subcommittees conducted SRMAs following the Preferred Reporting Items for Systematic Reviews and Meta-Analyses (PRISMA) or PRISMA-Diagnostic Test Accuracy (PRISMA-DTA) standards to ensure transparency and methodologic rigor.^69^, ^70^, ^71^, ^72^, ^73^, ^74^ Covidence, a web–based systematic-review platform, facilitated reference management, screening, and data extraction to ensure consistency across teams.^75^ Regular meetings promoted alignment in methodology, inclusion criteria, and interpretation of evidence.

These SRMAs form the evidence base for new GRADE–based clinical practice guidelines that will replace or refine specific recommendations from the original 2014 GED Guidelines. Unlike the original consensus-based recommendations, which did not assess the certainty of evidence or provide formal justification for recommendation strength, the new guidelines incorporate structured-evidence grading, consideration of patient values and preferences, health equity, and feasibility of implementation (Fig 4).^46^^,^^48^, ^49^, ^50^, ^51^, ^52^, ^53^ For example, the delirium guideline evaluates screening tools such as the Brief Confusion Assessment Method (bCAM) and the 4 A’s Test (4AT), along with risk stratification and ED management pathways, culminating in recommendations that balance diagnostic accuracy, workflow integration, and patient safety. Other guidelines—for medication safety, falls, and dementia—address key clinical questions such as which pharmacologic agents to avoid or prefer, effective fall risk screening and mitigation strategies, and approaches for dementia recognition and care coordination in the ED.

The GED 2.0 delirium clinical practice guidelines have been completed and will be submitted for publication following public review in 2025. Guidelines for medication safety, fall prevention, and dementia are in development, whereas the other domains are expected to be published in late 2025 or 2026.

### 1.7 Building Support and Dissemination Strategies

The GED Guidelines 2.0 working group is also focused on expanding broader organizational support to enhance dissemination and impact. Building on the original coalition of endorsing societies, the updated initiative seeks formal support from additional national organizations, including GEAR 2.0, the GED Collaborative, and other national multidisciplinary organizations. These partnerships help shape dissemination strategies, increase visibility, and foster adoption by aligning the guidelines with stakeholder priorities. Incorporating feedback from these groups supports knowledge sharing and plays a critical role in narrowing the research-to-practice gap in GEM.^51^^,^^76^

Seven SRMAs related to delirium, frailty, dementia, and medication safety have been published to date.^8^^,^^61^, ^62^, ^63^, ^64^^,^^67^^,^^68^ Preliminary findings from the GED Guidelines 2.0 initiative have also been presented at national EM meetings, offering early insights into potential changes to clinical practice recommendations.^65^^,^^66^ These early presentations have highlighted evidence gaps, confirmed strong support for certain practices (eg, use of validated delirium tools), and revealed areas in which guideline updates may challenge current norms.^8^^,^^61^^,^^62^ Notable findings include the effectiveness of ED-based programs in reducing potentially inappropriate medications and adverse drug events, the need for safer prescribing practices in the management of acute agitation, and the limited utility of routine head computed tomography in older adults with delirium or altered mental status unless specific risk factors are present.^63^^,^^64^, ^65^, ^66^ Work in frailty and dementia has further emphasized the value of structured assessments and highlighted how targeted interventions, such as community paramedicine and dementia–informed care models, can reduce revisits and hospitalizations.^67^^,^^68^ These early insights are directly informing the development of new clinical practice guidelines and help define future research and quality-improvement priorities.

The GEAR 2.0 website now serves as a central repository for GED Guidelines 2.0-related updates, enabling open access to publications, tools, and resources for clinicians, researchers, administrators, and policy makers.^31^ Anticipated dissemination activities include implementation workshops, educational webinars, podcasts, and tailored content for different clinical roles and settings.^77^^,^^78^ These efforts aim to improve guideline uptake across both GED-accredited and nonaccredited EDs. Social media campaigns and hospital-based outreach will further support public and institutional awareness in advance of guideline release.

### 1.8 GED Guidelines 2.0 Lessons Learned

The development of the GED Guidelines 2.0 has highlighted important lessons in stakeholder engagement, project management, and collaboration. Early involvement of stakeholders helped align priorities and goals, which has been valuable in securing organizational support and will be essential during the dissemination and implementation phases. Smaller, PICO-specific teams streamlined the SRMA process by distributing workloads and integrating clinical and research perspectives. This structure improved collaboration and expedited the progress across the 7 priority topic subcommittees.

The creation of GRADE–guided clinical practice guidelines requires time, patience, and sustained commitment. Although most working group members brought academic and research experience, there was variability in familiarity with systematic-review methods and GRADE-specific processes. This required ongoing education, recalibration of workflows, and in some cases, shifting roles to meet evolving project needs. These challenges highlight the value of multidisciplinary collaboration, particularly the important roles played by methodologists, medical librarians, statisticians, and content experts, in upholding methodologic rigor.

At the heart of this initiative has been a deep culture of academic volunteerism. The GED Guidelines 2.0 effort was made possible by a dedicated, mission-driven group of academic geriatric and EM clinicians who collectively volunteered thousands of hours to progress this work. This initiative has been sustained not only by academic output, but by shared purpose, collegiality, and a deep commitment to advancing care for older adults. That foundation helped carry the project through the COVID-19 pandemic and continues to drive progress today.

### 1.9 Guiding the Future: Anticipated Impact of the GED Guidelines 2.0

Emergency physicians place the highest value on clinical practice guidelines with actionable recommendations, explicit target populations, and explicit links between the strength of evidence to support recommendations and anticipated outcomes.^38^ The updated GED Guidelines 2.0 provide these attributes while offering practical strategies that can be implemented across a wide range of ED settings and transparently weighing the anticipated benefits, potential harms, resource requirements, acceptability, feasibility, and health equity. Many successful geriatric emergency care models, especially those expanded during the COVID-19 pandemic, demonstrate that hospitals can improve care for older adults without necessarily pursuing full GED accreditation.^42^^,^^79^, ^80^, ^81^ Instead, localized solutions such as dedicated geriatric champions, protocol–based care pathways, and specialized practitioner roles allow EDs to apply core GED principles in ways that reflect each institution’s resources and priorities.^24^^,^^82^ This flexible approach supports broader adoption of foundational practices including screening, assessment, and patient-centered care, enabling EDs of all types to enhance care pathways for older patients. These models have also been associated with reduced healthcare utilization, lower costs, and improved outcomes for older adults and their caregivers.^83^, ^84^, ^85^, ^86^

The GED Guidelines 2.0 aim to build on this momentum, equipping EDs with a more transparent, introspective, and practical framework to guide decision making, foster innovation, and advance geriatric emergency care delivery nationally. A key strength of the initiative lies in its foundation of rigorous evidence synthesis. Initial SRMAs conducted by the GED Guidelines 2.0 working group have already identified opportunities to improve geriatric ED care.^61^^,^^63^^,^^64^^,^^67^^,^^68^ Despite this momentum, gaps remain. The long-term effects of GED model implementation—particularly on outcomes prioritized by older adults such as functional status, mobility, and quality of life—are still not well understood.^87^^,^^88^ Existing research primarily focused on administrative metrics such as ED revisits and readmissions, rather than holistic, person-centered outcomes.^87^, ^88^, ^89^ Addressing this evidence gap will require ongoing investment in quality improvement, prospective studies, and randomized trials to fully assess the real-world effects of GED Guidelines 2.0 implementation.

## 2 Conclusion

Although the GED Guidelines 2.0 clinical practice guidelines and related implementation efforts are still in development, sharing the methodology, vision, and anticipated outputs is a crucial first step toward promoting widespread adoption. By building upon the original guidelines, the GED Guidelines 2.0 strives to advance geriatric emergency care through a flexible, evidence-informed framework grounded in multidisciplinary collaboration and transparency. This initiative underscores not only the importance of improving care for older adults but also the feasibility of doing so across diverse clinical environments even while awaiting higher-quality, less-biased proof-of-concept research. As we continue to finalize and release the new guidelines, we offer a roadmap for other EM subspecialties to create high-impact, GRADE–based clinical practice guidelines tailored to their unique areas of care. Through transparency, innovation, and shared purpose, the GED Guidelines 2.0 aims to strengthen the science and practice of GEM and improve outcomes for older adults across the healthcare continuum.

## Funding and Support

This work was funded and supported by the 10.13039/100000909John A. Hartford Foundation and the West Health Institute, aimed at advancing the Geriatric Emergency Department Collaborative and the development of the Geriatric Emergency Department Guidelines 2.0.

Satheesh Gunaga, Christopher R. Carpenter, Maura Kennedy, Lauren T. Southerland, Alexander X. Lo, Sangil Lee, Fabrice Mowbray, Rachel M. Skains, Teresita M. Hogan, Martin F. Casey, Kei Ouchi, Naomi R. George, Cameron J. Gettel, Katherine Selman, Luna C. Ragsdale, Anita N. Chary, James D. van Oppen, Glenn Arendts, Charles L. Maddow, Katherine M. Hunold, Katren R. Tyler, Danya Khoujah, Ula Hwang, and Shan Liu report financial support was provided by The John A. Hartford Foundation and West Health Institute.

## Declaration of Generative AI and AI-Assisted Technologies in the Writing Process

During the preparation of this work, the authors used ChatGPT (OpenAI) in order to enhance grammar, improve language clarity, and support the readability of the manuscript. After using this tool, the authors reviewed and edited the content as needed and take full responsibility for the content of the publication.

## Conflict of Interest

Satheesh Gunaga is a volunteer board member for Compassion and Choices, a non-profit organization. Satheesh Gunaga recieved funding as a site Sub-Investigator on an NIH-funded study (NIH Prime Award No. 1U19AG078105-01A1) during the conduct of this initiative. Christopher R. Carpenter was awarded grants from the National Institute on Aging (NIA) through R33AG058926 and R61AG069822, the John A. Hartford Foundation, and the West Health Institute. Christopher R. Carpenter held leadership positions with the Geriatric Emergency Care Applied Research (GEAR) Network, the Clinician-Scientists in Transdisciplinary Aging Research (Clin-STAR) Coordinating Center, and the ACEP Geriatric Emergency Department Accreditation Advisory. Maura Kennedy received funding from the American College of Emergency Physicians and Gillian Reny Stepping Strong for Trauma Innovation, Brigham Health. Lauren T. Southerland was awarded grant from the NIA through K23AG061284. Rachel M. Skains was awarded grants from the NIA (R33AG058926) and the West Health Institute. Cameron J. Gettel was the pepper scholar with support from the Claude D. Pepper Older Americans Independence Center at Yale School of Medicine (P30AG021342) and the NIA (R03AG073988). Ula Hwang was awarded grants from the NIA (R33AG058926, R33AG069822), the John A. Hartford Foundation, and the West Health Institute. Shan Liu was awarded grants from Gillian Reny Stepping Strong for Trauma Innovation, Brigham Health; the John A. Hartford Foundation; and the West Health Institute.
